# Supplementary material for: Designing school reopening in the COVID-19 pre-vaccination period in Bogotá, Colombia: A modeling study
Source: PLOS Glob Public Health. 2022 Jun 15;2(6):e0000467. doi: 10.1371/journal.pgph.0000467 (PMC10021412; doi:10.1371/journal.pgph.0000467)
Supplement: S1 Text — (DOCX) [file pgph.0000467.s001.docx]

# Supplementary material


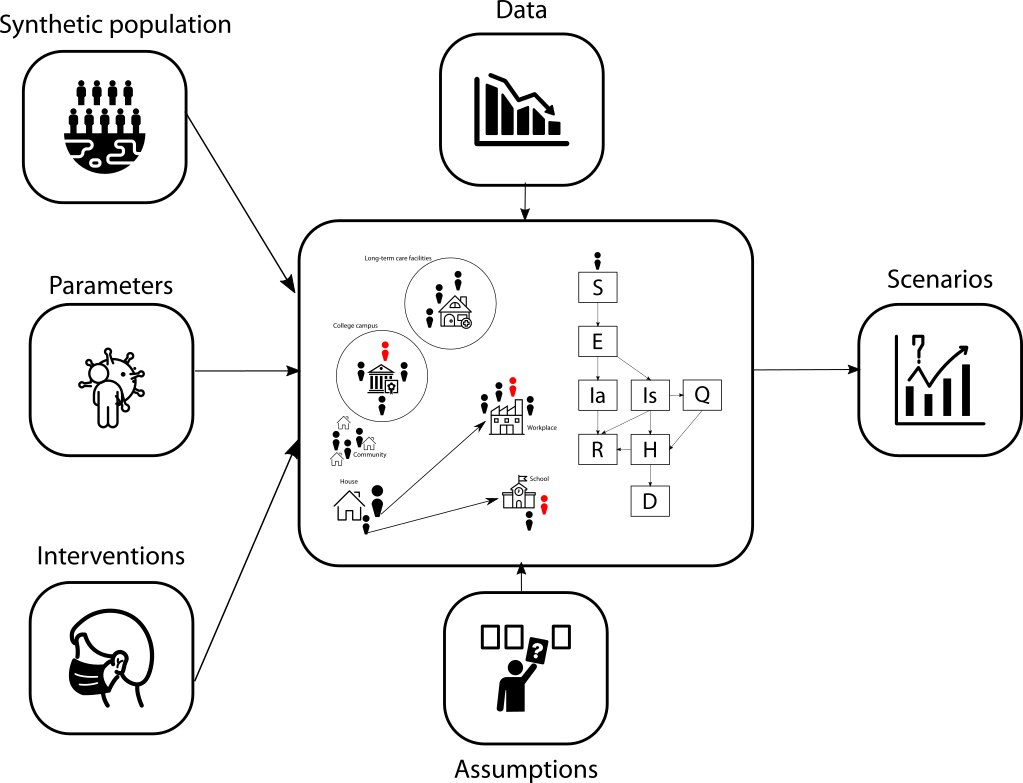


Fig A. Description of agent-based model.


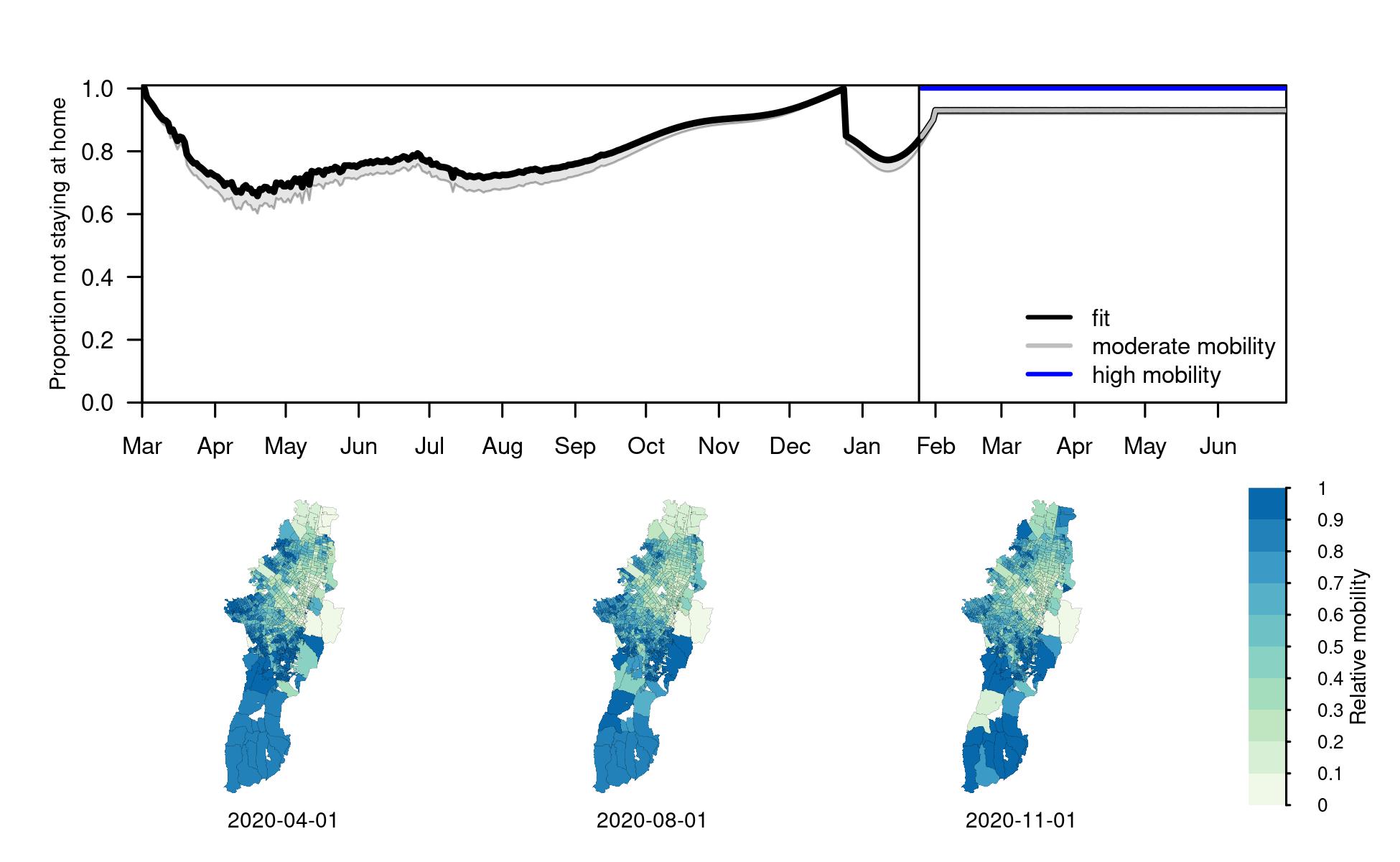


Fig B. Adjusted mobility in time and space for the city of Bogotá. Top panel shows the calibrated reduction in mobility over time. The black line shows the mobility in the model fit to the data, the blue line shows higher mobility, and the gray line shows moderate mobility. The bottom panel shows the geographical distribution of mobility restrictions at three different time points. In this panel, darker colors represent more mobility, and lighter colors represent less mobility. The base layer for these maps were taken from the datos abiertos dataset (<https://datosabiertos.bogota.gov.co/dataset/sector-catastral>).


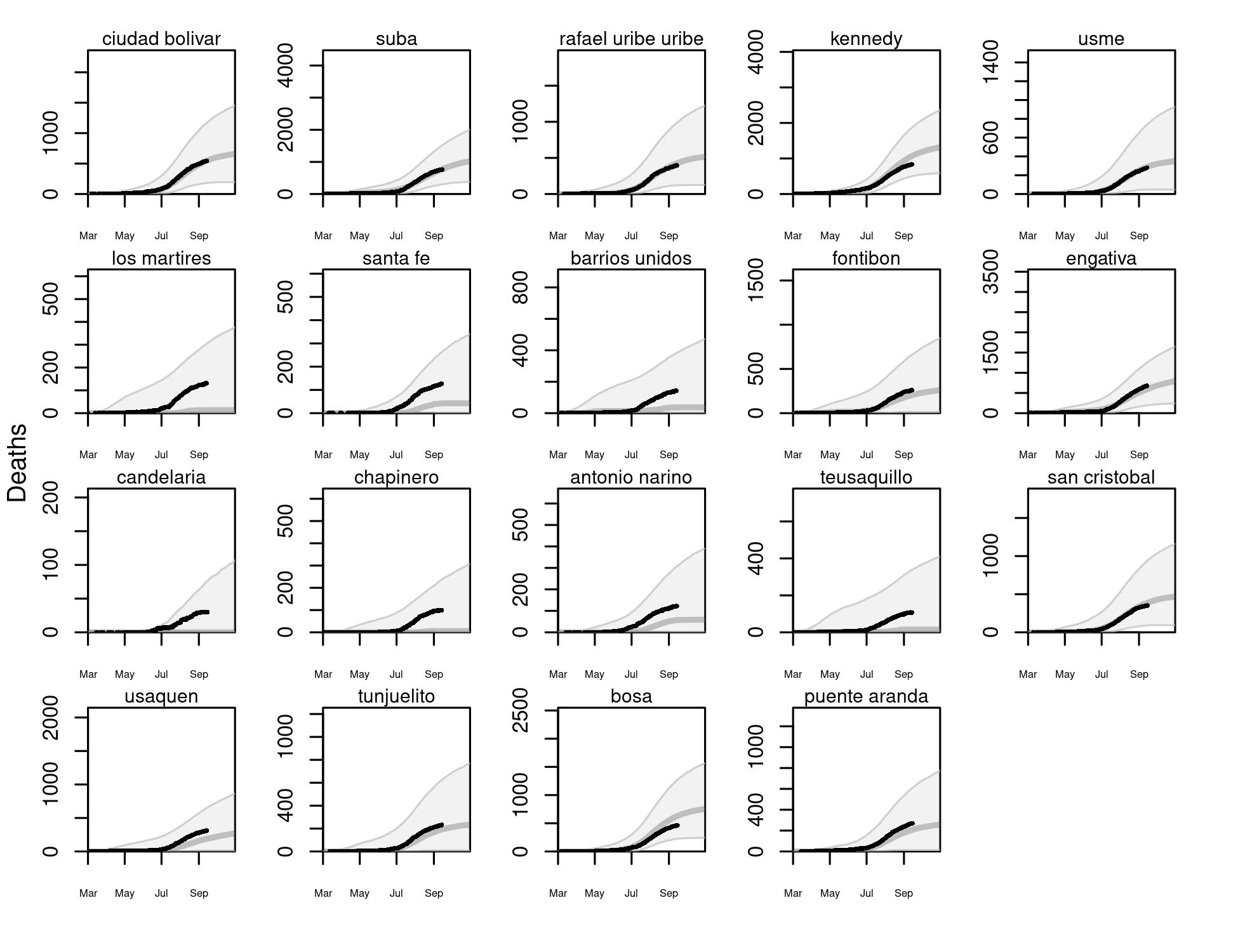


Fig C. Model comparison with data excluded from calibration for each locality. Each panel shows the daily cumulative incidence of deaths for each locality in Bogotá, Colombia. Black line represents the official data, and gray lines and shaded areas represent the model median estimates and 95% CrI. Assumption of lower (50%) susceptibility in <10 years.


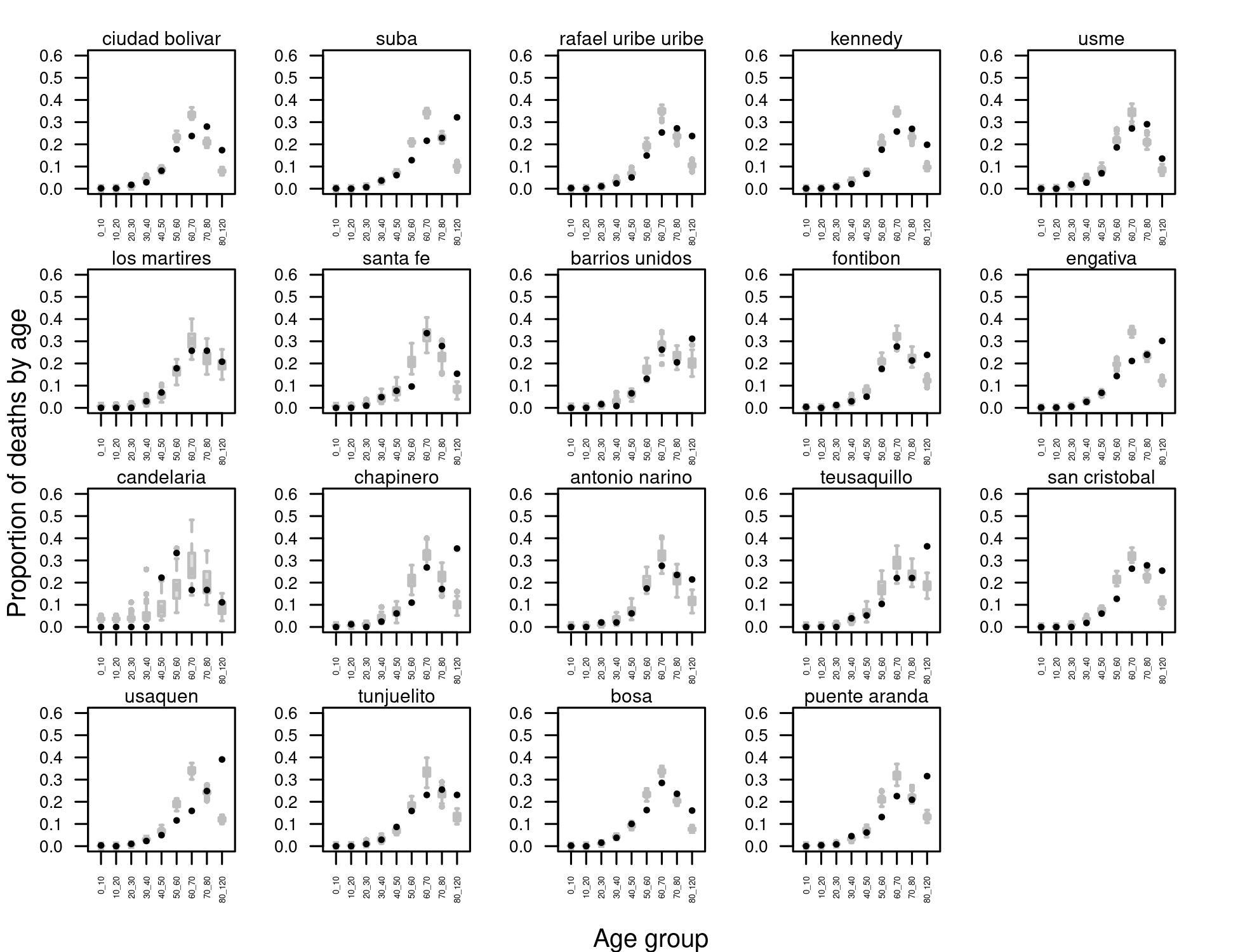


Fig D. Model comparison with age-specific data excluded from calibration for each locality. Each panel shows the proportion of total deaths by each age group in each locality in Bogotá, Colombia. Black points show the data and gray box plots show the model estimates. Assumption of lower (50%) susceptibility in <10 years.


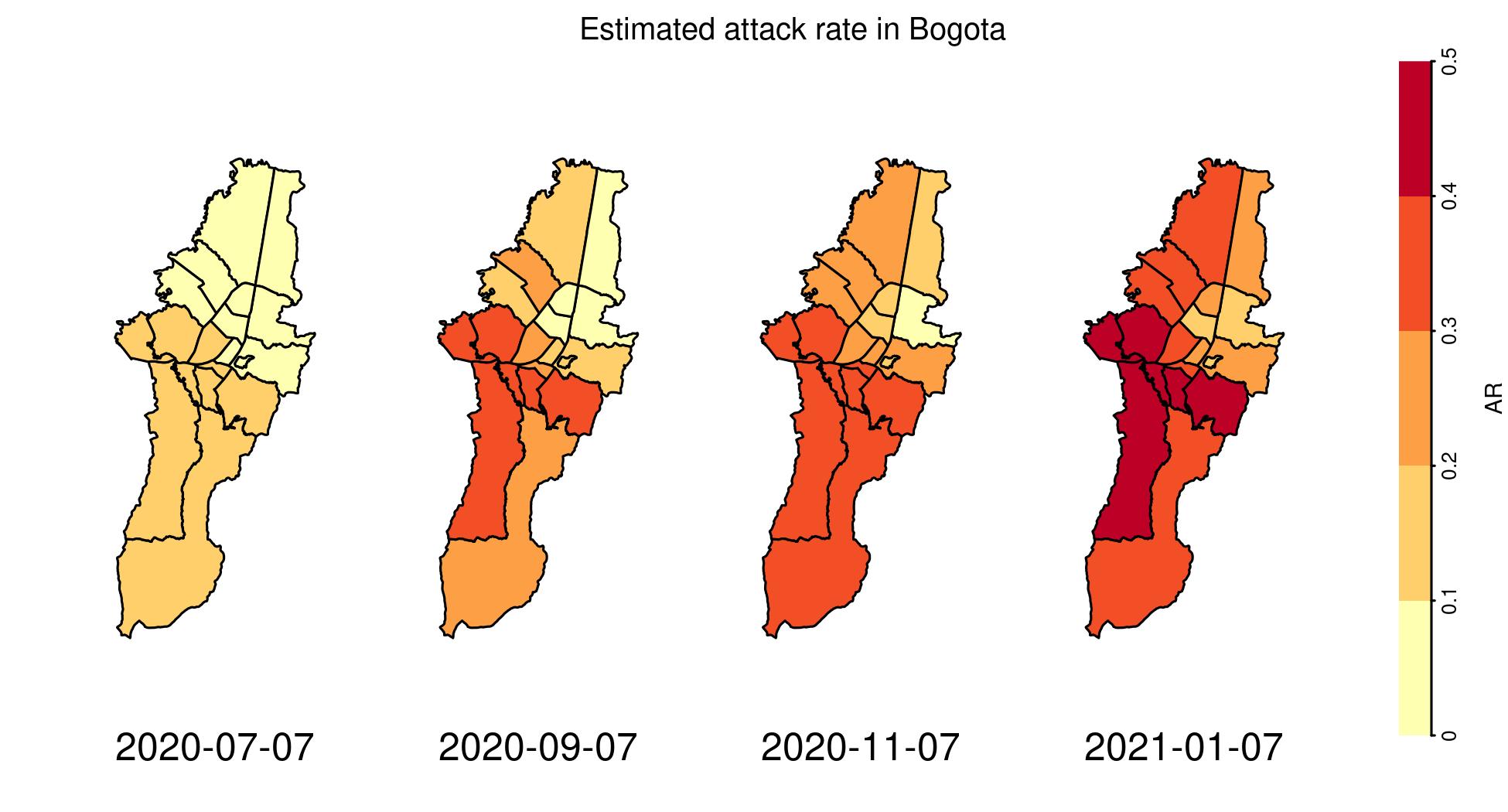


Fig E. Model estimates of attack rate by locality in Bogotá at different times points. Darker red colors represent a higher proportion of the population infected by each date, and lighter yellow colors represent a lower proportion of the population infected by each date. Assumption of lower (50%) susceptibility in <10 years. The base layer for these maps were taken from the datos abiertos dataset (<https://datosabiertos.bogota.gov.co/dataset/sector-catastral>).


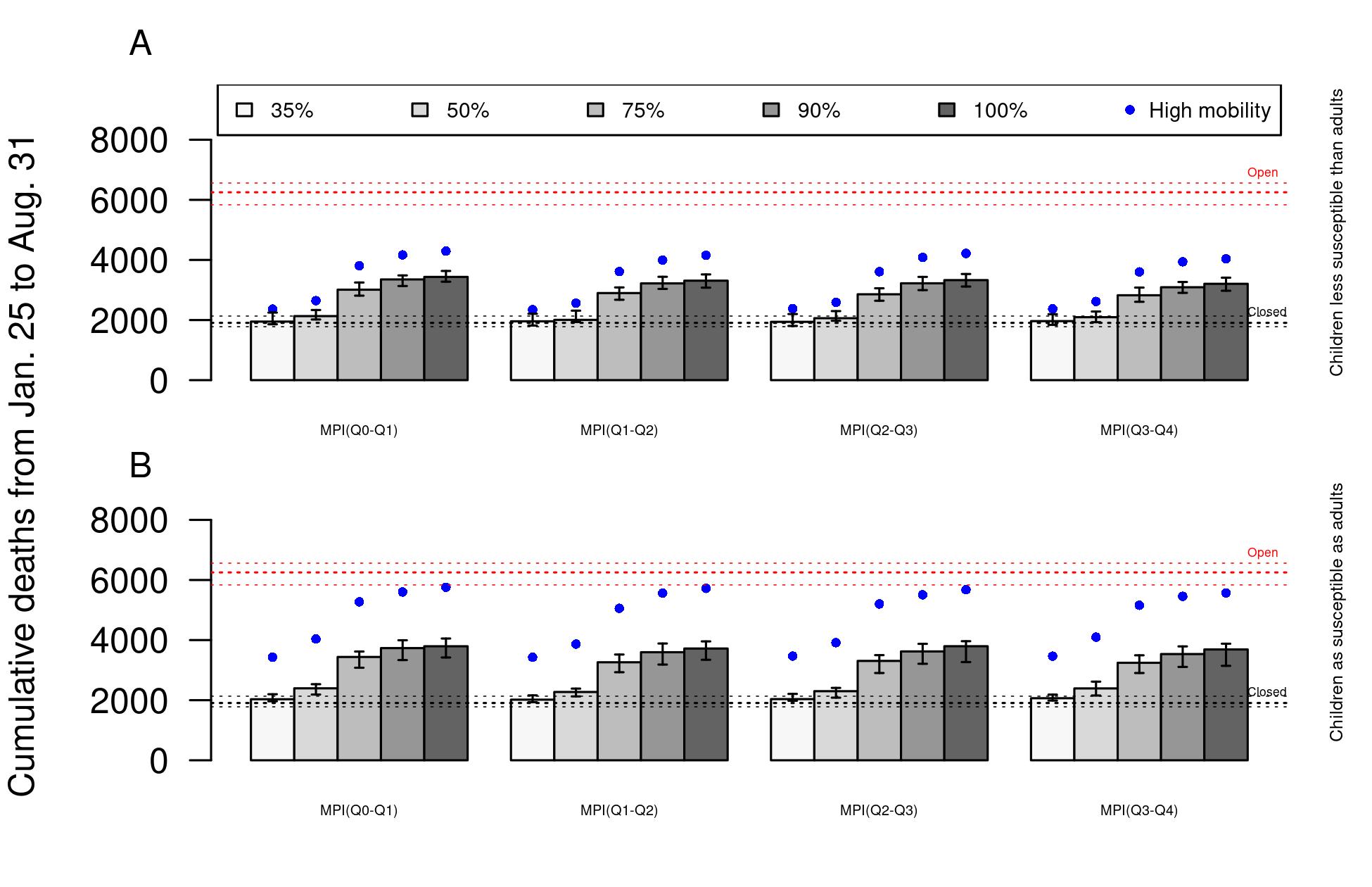


Fig F. Total cumulative deaths under different school reopening strategies from January 25 to August 31, 2021. Cumulative deaths of scenarios in which schools reopen by multidimensional poverty index of schools. A) Assumption of lower susceptibility for <10 years. B) Assumption of equal susceptibility to infection for all ages. In all scenarios, red dotted lines show the median and 95% CrI of the scenario with full schools reopening (with face masks), and the black dotted line shows the median and 95% CrI of the scenario with all schools closed. Blue dots show a scenario with high mobility when schools reopen. In all scenarios, we assumed long-term protection after SARS-CoV-2 infection.


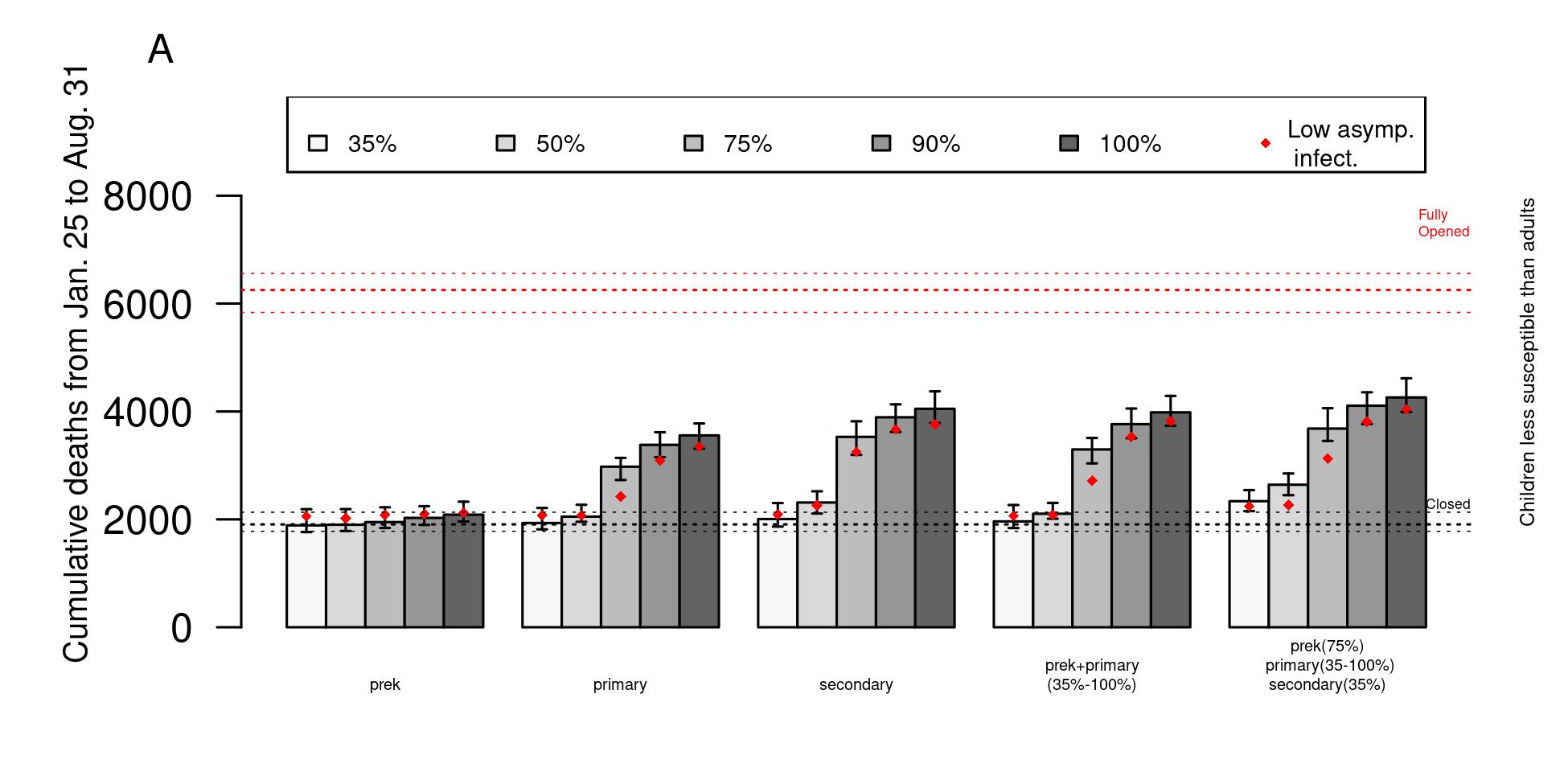


Fig G. The impact of lower asymptomatic infectiousness in the total cumulative deaths under different school reopening strategies from January 25 to August 31, 2021. A) Cumulative deaths of scenarios in which schools reopen by grades with an assumption of lower (50%) susceptibility in <10 years. From left to right, the first group of bars show exclusive reopening by grade groups in which the other grades remain closed. The fourth group of bars (pre-K+primary) represents a scenario in which pre-K primary and primary reopen at different capacities but secondary remains closed. The last group shows a scenario in which all grades go to in-person school at some level, with pre-K fixed at 75%, secondary fixed at 35%, and primary varying from 35% to 100%. Red diamonds show the median estimate of the same scenario with 75% infectiousness of asymptomatics. In all scenarios, we assumed long-term protection after SARS-CoV-2 infection and lower (50%) susceptibility in <10 years.


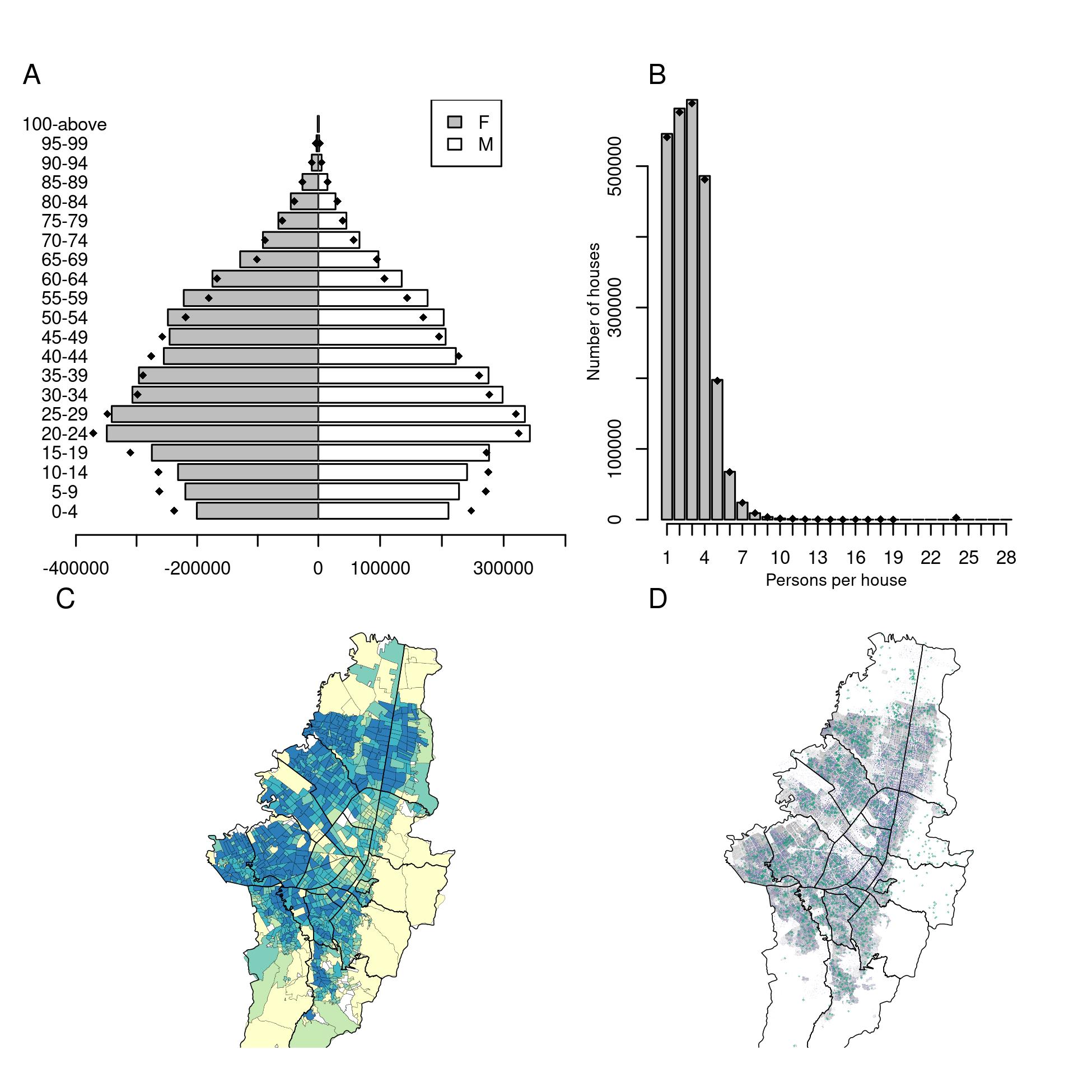


Fig H. Synthetic population of Bogotá, Colombia. A) Comparison of aggregated population by age and sex. Bars show the synthetic population and black dots the census data from Bogotá, Colombia. B) Household distribution for the synthetic population (bars) and data(points). C) Population density by Census Tract (Unidad Catastral). Darker colors show higher population density. D) Location of households (gray), schools (green), and workplaces (purple) in the synthetic population of Bogotá. The base layer for these maps were taken from the datos abiertos dataset (<https://datosabiertos.bogota.gov.co/dataset/sector-catastral>).


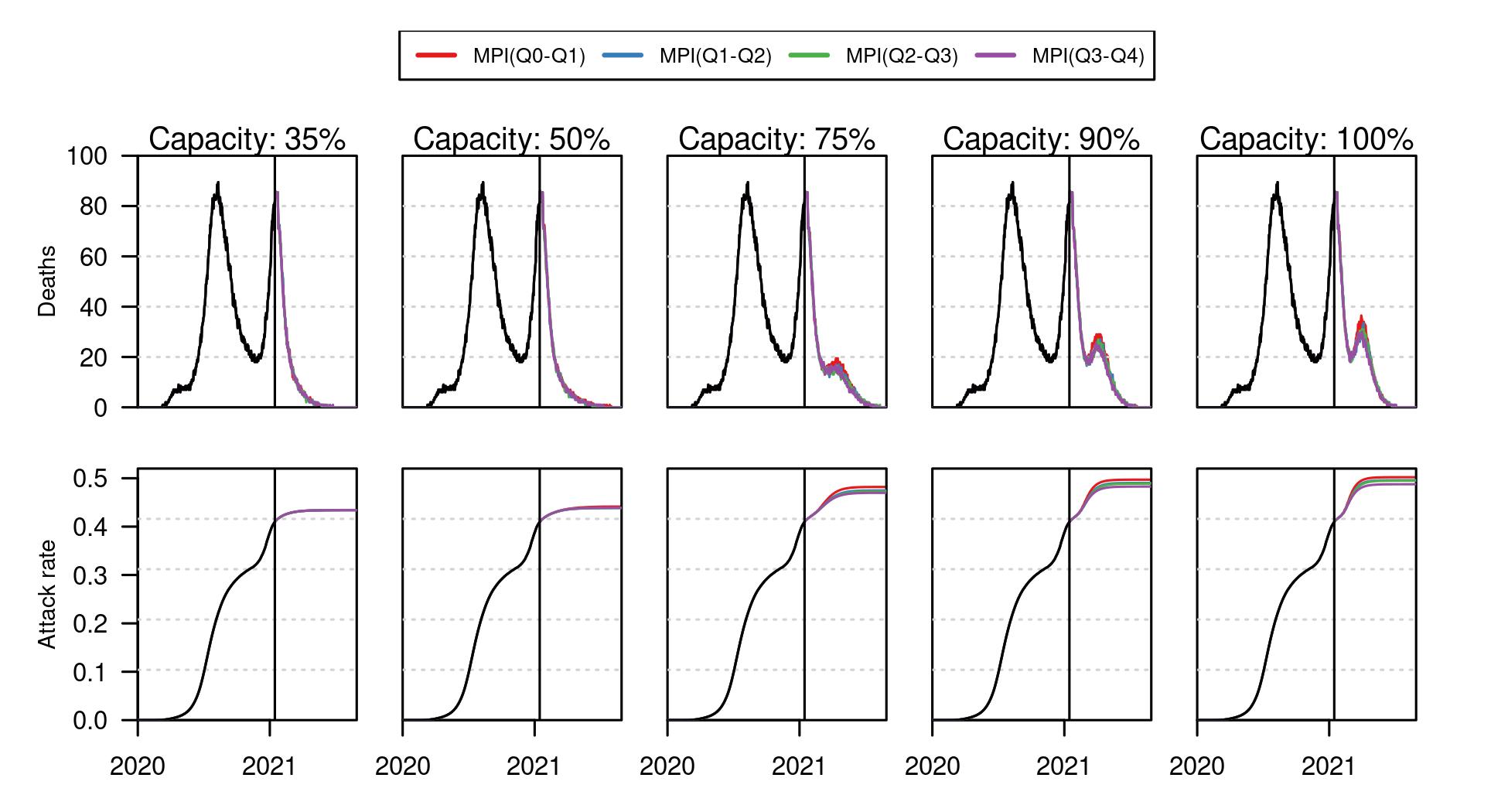


Fig I. The impact of delaying school reopening by multidimensional poverty index of schools. We assumed lower (50%) susceptibility in <10 years. Each column shows a different capacity level. Each group of MPI shows a set of schools grouped according to their MPI. Bottom panel shows the median estimate of attack rates for each scenario. Vertical black line shows the initial date of school reopening (January 25, 2021). All scenarios were simulated until August 31, 2021. Black lines show the model outputs before the simulated date of school reopening.


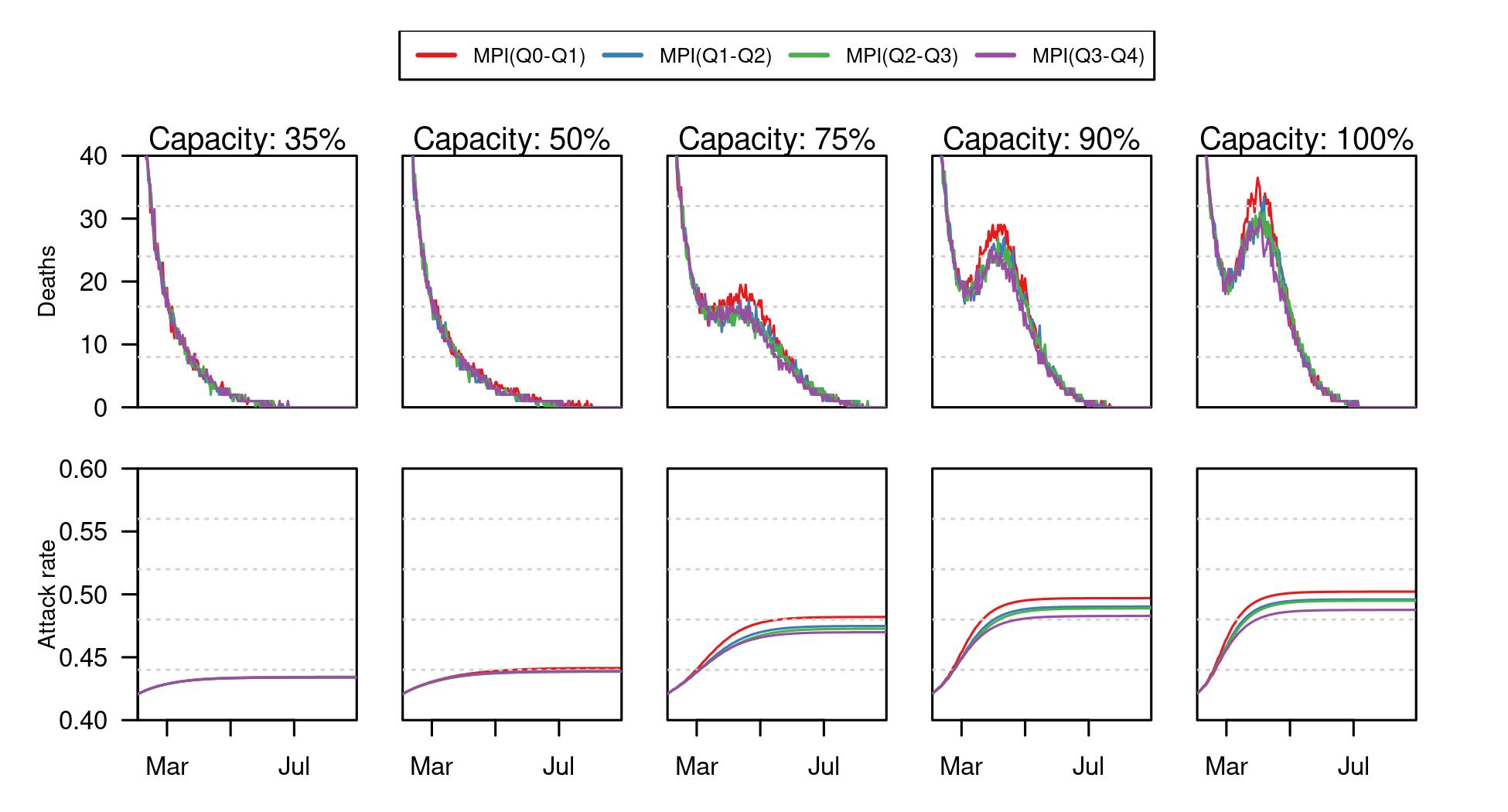


Fig J. Zoom of the impact of delaying school reopening by multidimensional poverty index of schools. We assumed lower (50%) susceptibility in <10 years. Each column shows a different capacity level. Each group of MPI shows a set of schools grouped according to their MPI. Bottom panel shows the median estimate of attack rates for each scenario. Vertical black line shows the initial date of school reopening (January 25, 2021). All scenarios were simulated until August 31, 2021.


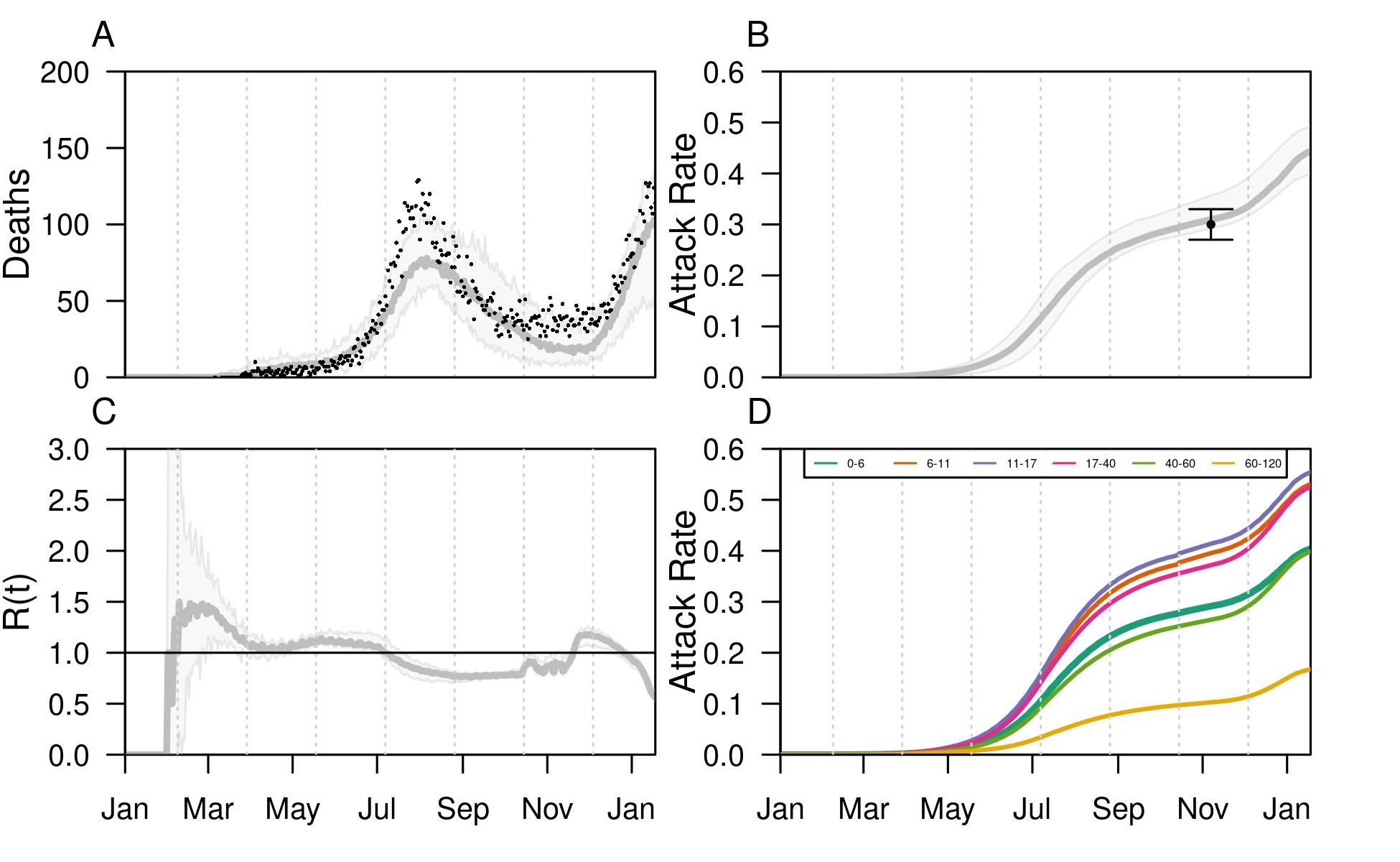


Fig K. Model fit to data with assumption of equal susceptibility for all ages. A) Model fit to daily incidence of deaths. Black dots show the official data, and gray lines show the median estimate of the model with the 95% CrI represented by gray-shaded curves. B) Model estimates of attack rate in time represented by gray line (median) and shaded area (95% CrI). The point and arrows show the median estimates and CI of official serological study in Bogotá. C) Estimated reproduction number in time. D) Estimated attack rate in time for different age groups.


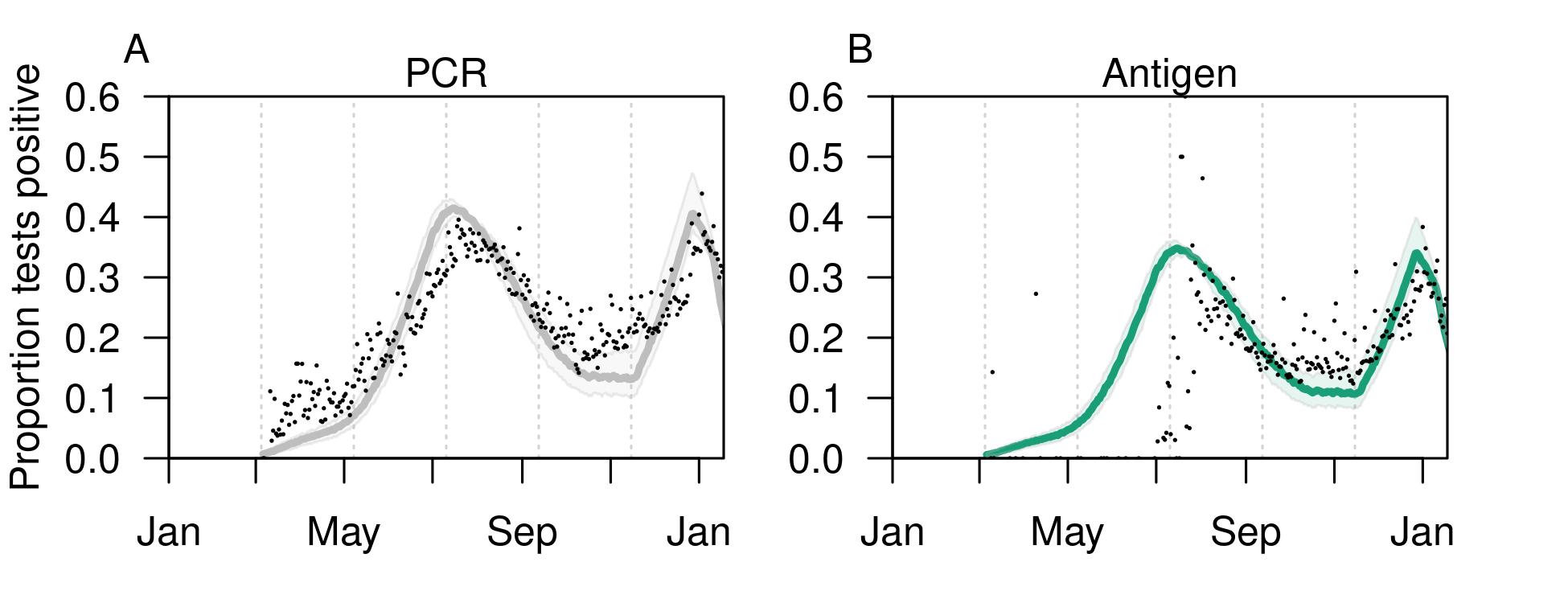


Fig L. Model comparison to test positive data. We assumed lower (50%) susceptibility in <10 years. A) daily proportion of PCR tests positive by date of sample. Gray line and shaded area show the median and 95% CrI of the model simulations. Black points show the data. B) daily proportion of antigen tests positive by date of sample. Gray line and shaded area show the median and 95% CrI of the model simulations. Black points show the data.


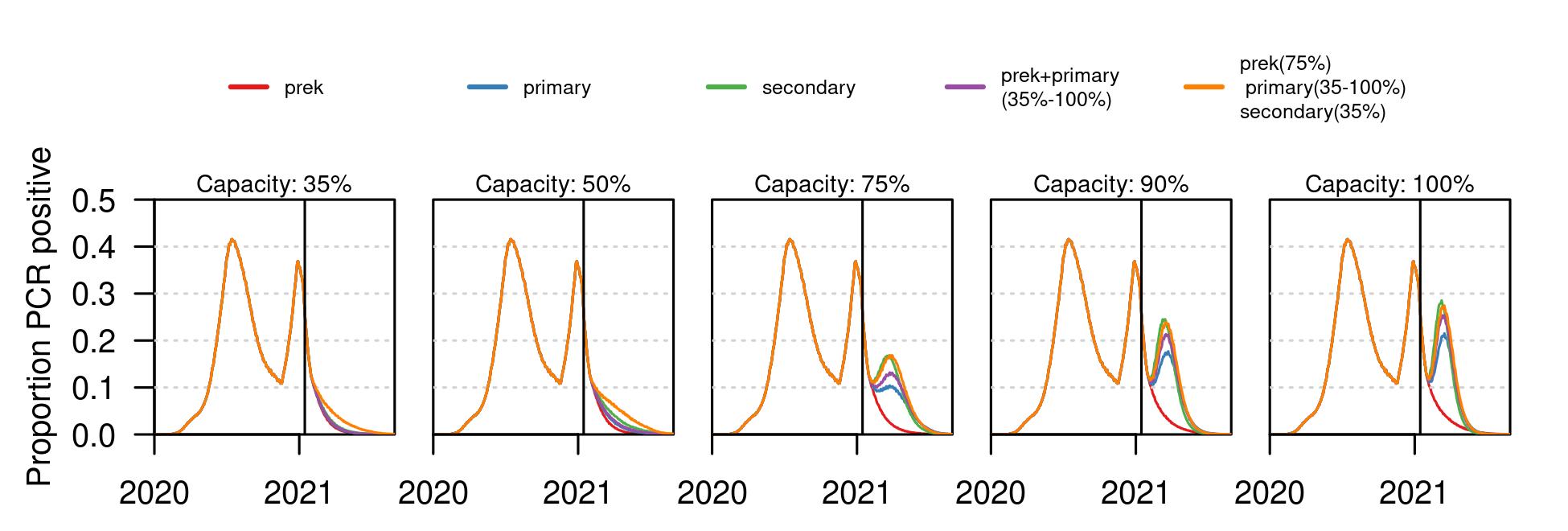


Fig M. The impact of school reopening on the positive rate of PCR testing for different grades in in-person school. Assumption of lower (50%) susceptibility in <10 years. Each column shows a different capacity, and each line shows a different strategy of school reopening base on grades. Black vertical line shows the baseline date of school reopening.


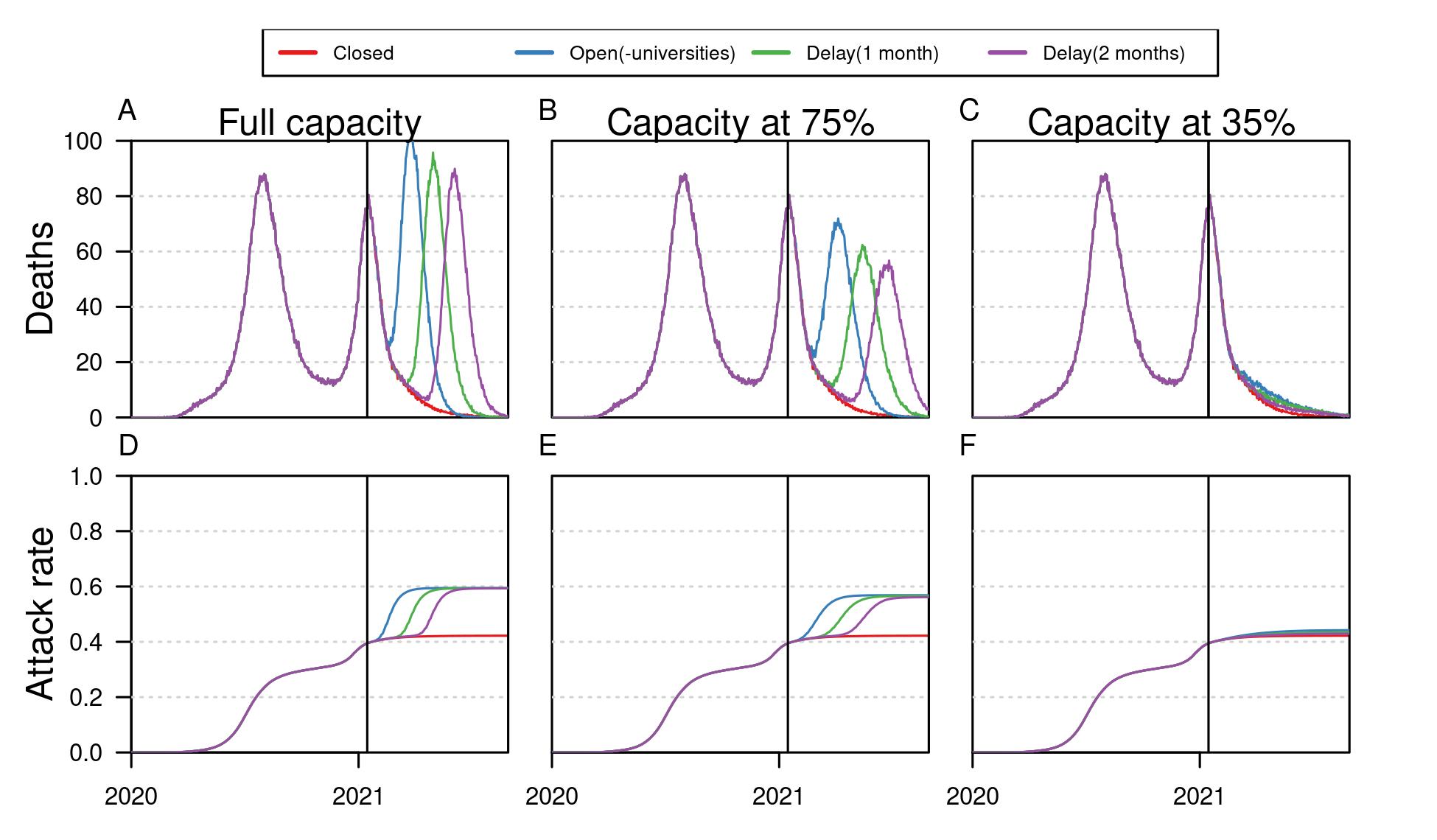


Fig N. The impact of delaying school reopening. Each column shows a different capacity level. Red lines represent a scenario in which all schools remain closed, blue lines represent K-12 schools open, green and purple lines show scenarios of delaying school reopening by 1 and 2 months, respectively. Top panel shows the median estimate of daily incidence of deaths. Bottom panel shows the median estimate of attack rates for each scenario. Vertical black line shows the initial date of school reopening (January 25, 2021). In these scenarios, we assumed equal susceptibility for all ages.
