# Supplementary material for: Designing school reopening in the COVID-19 pre-vaccination period in Bogotá, Colombia: A modeling study
Source: PLOS Glob Public Health. 2022 Jun 15;2(6):e0000467. doi: 10.1371/journal.pgph.0000467 (PMC10021412; doi:10.1371/journal.pgph.0000467)
Supplement: Attachment — Submitted filename: manuscript_bogota_school_reopening_response_to_reviewers_R1.pdf [file pgph.0000467.s002.pdf]

Response to Reviewers

We thank the reviewers for their thoughtful and constructive comments. We have addressed each of the comments and have elaborated a response for them. Please see below our point by point response for each of the reviewers' concerns with line numbers referring to the PDF version of the report with changes marked in blue(additions) and red(deletions).

Reviewer 1

1. *Agent-based models can be notoriously difficult to understand. It would be helpful to readers if the authors could provide a high-level summary of the model. I agree that referring interested readers to Guido et al 2020 is appropriate given its excellent account of technical details, but a simple schematic may help the article reach a wider audience. In particular, the summary should help one understand how various data sources feed into the model and where key assumptions may affect model outputs (e.g. deaths and attack rates).*

   Response: We have added a schematic to the supplementary material summarizing the model and how the data is used in the model (Line **505**).

2. *The authors give a proper introduction as to the relevance of school reopening and its impact to public health in Bogota, Colombia. However, the rationale for the methodology is not addressed. For example, what is the benefit of using this stochastic agent-based model vs a simpler stochastic/deterministic compartmental model? Adding 1-2 sentences somewhere in the paragraph beginning at Line 81 would suffice.*

   Response: We now included a couple of sentences to explain that agent-based models could incorporate heterogeneous dynamics of transmission (Lines **73** - **80**).

   "The transmission of SARS-CoV-2 can be heterogeneous across demographic and geographic characteristics of the population. For instance, early non-pharmaceutical interventions implemented to curb the impact of COVID-19 required the ability of people to stay at home for a prolonged period, creating heterogeneous contact patterns in the population, with a potentially higher contact rate in lower income settings. In contrast to compartmental models, agent-based models are capable of incorporating different levels of heterogeneity in transmission due to various factors, such as contact rates or adherence to public health interventions"

3. *The authors explore a wide range of scenarios for reopening but do not discuss why these scenarios (e.g. opening schools only for younger children) were chosen. Given the nature of the journal, I feel it is appropriate for the authors to expand why these scenarios are pertinent to Bogota and whether they relate to any public health policies discussed/implemented in Colombia as a whole.*

   Response: The scenarios that we considered in this study were designed to inform decision makers on the impact of school reopening on COVID-19 dynamics in the city. We had frequent discussions with the Secretary of Health of Bogotá to design these experiments. We have explained this in lines **607** - **615**.

4. *In Figure S2, model estimates of deaths closely align with data in most localities. In particular, the sudden uptick around Jun/July 2020 is recapitulated. However, two localities stick out, Chapinero and Teusaquillo, where (i) quantitatively, estimates and data diverge in terms of magnitude in deaths, and (ii) qualitatively, the surge never occurs in the median trajectories. Yet, the 95% CrIs do capture the shape of the data. Can the authors comment on why the model does not predict a median trajectory of similar shape to the data? Interestingly, proportions of deaths (Figure S3) are underestimated in older populations (80-120) in both localities pointed out, as well as others.*

   Response: This is an important aspect of the model that we are aware of. We have unsuccessfully tried to improve the fit in these localities for many months. We have concluded that we could be underestimating the contact rates of elders in these localities because of incomplete data on nursing homes, or other similar places. We now expanded our description of the results to explicitly explain how the model underestimates the number of deaths in these localities (Lines **104** - **106**). Similarly, we acknowledge this limiation on the discussion section (Lines **454** - **459**).

5. *Based on Figure S5, MPI and deaths do not seem to be correlated. That is, no MPI subgroup seems to be dispproportianately burdened by deaths in simulations across scenarios and assumptions about susceptibility. Are there any model outputs that seem affected by MPI? If not, what role does MPI play in the simulation? This is alluded to in the discussion, but not quantified. Is it possible to illustrate how these subgroups are distributed over geography?*

   Response: A key point of the model is that we do not have a cut-point for poverty related to MPI. so we tested whether the distribution of MPI by quantiles would have an impact on the burden of COVID-19. The model shows there is no impact just based on this particular distribution regardless of the geographical distribution of schools. The key message here is that the impact does not come from the empirical distribution of MPI itself.

6. *The important conclusion at Lines 204-206 is a little difficult to understand. The level of community contact (based on mobility) increases deaths across all scenarios, but what exactly constitutes an acceptable burden of cases? That is a difficult philosophical question that modelling alone cannot answer, and it is not clear how it *should* be addressed. If it is not too difficult, the authors may be able to examine how the additional deaths due to reopening are distributed across localities and schools. One could argue that a distribution where deaths concentrate in a locality or around a particular school indicates an unfavorable/unjust/riskier scenario for a society compared to a more randomly distributed pattern.*

   Response: Our intent with that sentence was to argue that schools would not have been the only cause of a new wave of COVID-19 in the city, and that other aspects, such as increased community contact should also be considered. We now restructured this sentence to make it more clear (Lines **219** - **230**).

   We agree with the reviewer that this is a very difficult question that modeling should not answer alone. Our metric was decided as the impact on the overall COVID-19 burden in

the city, instead of individual communities given that the main risk at the time of this study was to overwhelm the public health sector. Here, we present the different levels of increased risk at the city level due only to reopening of schools. We then allowed the Secretary of Health and the Secretary of Education to determine what would be acceptable from a city-wide perspective. We have expanded our discussion to make this clear in lines **219 - 230**.

7. *The effect over time of reopening at or near full capacity is quite stark, even when only primary schools reopen and at a lower susceptibility in children. Can the authors confirm that this effect in simulations is strictly due to reopening? Notably, the population pyramid in Figure S7A indicates that the synthetic population has fewer children/young adults under 20 compared to the census data. This is not necessary for the main text, but it would be informative to somehow quantify the model's sensitivity to the size of this demographic in this specific scenario. Weak or lack of sensitivity would suggest that the contact patterns in these groups are driving the surge.*

   Response: To isolate the effect of reopening schools from the rest of the city, we simulated various scenarios of transmission with and without school reopening in which we increased mobility and contacts in the city. Our results suggest that the isolated effect of reopening schools would have an effect of increased transmission in different scenarios of contact and mobility outside the schools. However, reopening schools does not only result in outbreaks inside schools, given that infections that start in schools could eventually spread to the households of students and teachers, as well as the community overall.

   The reviewer is correct that the model underestimates the population under 20 compared to the census data. Even though it would be interesting to measure this sensitivity of the model to changes in the synthetic population, it would require to fit a different synthetic population to the census data, which would change geographic patterns and contacts overall. We included this limitation of the model in the discussion to acknowledge that our model could underestimate the infections in these ages, that could result in an overall underestimation of the total number of infections in those ages (Lines **459 - 462**).

8. *Finally, the authors should spend some time improving the prose of the text to improve its flow and clarity.*

   Response: We have made many changes throughout the manuscript, particularly in the results and discussion sections.

9. *The caption on Figure S1 states blue is used to indicate lower mobility but the figure legend uses blue for higher mobility.*

   Response: We have now modified the caption to address this comment.

10. *In the main text there are a few references to Figure S5 where I believe the intended reference is Figure S6.*

    Response: We revised this and found that all references to Figure S5 were as they were intended. Figure S5 is related to the impact of school reopening by schools' MPI at different

levels of susceptibility in children. We mentioned first in line **233** referring to reopening strategies based on income and the impact of susceptibility on the estimates. The second time we referenced this figure is in line **199**, where we discuss the effect of the multidimensional poverty index of schools on the number of deaths. Finally, we refer to this figure in lines **213** - **214**. We updated this sentence to clarify that we are referring to reopening strategies by grade and MPI.

11. *Figure S8 demonstrates a third surge due to increase capacity in reopening schools. It would be nice to zoom in on one of these examples to be able to see disparities in the different MPI groups since they are completely overlapping. A reference in the text would also suffice.*

    Response: We have added a new figure zooming in the period where schools reopen for the strategies based on MPI groups.

12. *At line 195, I would subsititute "insensitive" for "resilient" because the observed effect is not an innate property of areas with vulnerable populations.*

    Response: We now have made the change from resilient to insensitive (line **209**).

Reviewer 2

1. *Although compartmental mathematical models have been an essential tool in analyzing and forecasting pandemic trajectories, one should be aware of the limitations. A general discussion is, for instance, in Roda et al.: Why is it difficult to accurately predict the COVID-19 epidemic?, Infect Dis Model. 2020; 5: 271–281. For the specific case of schools one relevant analysis is in Gandolfi et al. "A new threshold reveals the uncertainty about the effect of school opening on diffusion of Covid-19." arXiv preprint arXiv:2104.04136 (2021). These issues should be addressed by the authors, at the very least in the "Limitations" section, but possibly relating to the results of the paper to the above references in other sections.*

    Response: We would like to point out that this is not a compartmental model. For instance, one of the strengths of agent-based models when compared to compartmental models is that agent-based models can incorporate different levels of heterogeneity in transmission. We now explicitly make that comparison in the lines **73** - **80**. We also included in the limitations section that we were not trying to forecast or predict the impact of COVID-19 in the city. Instead, we were evaluating hypothetical scenarios to understand the potential impact of school reopening by using a detailed agent-based model that incorporates multiple data types to calibrate the model to the current dynamics in the city. Our model is not setup to predict the course of the epidemic, but instead to evaluate the impact of different interventions (Lines **435** - **445**).

2. *1(b) The research relies on mortality data, so, in addition to the instabilities mentioned in 1(a), one has also an issue of data reliability. For instance, Karlinsky et al.: Tracking excess mortality across countries during the COVID-19 pandemic with the World Mortality*

Dataset, eLife 2021;10:e69336, indicates that mortality was underestimated in Bolivia by a factor of 1.2. This should also be addressed, at least in the "Limitations" section.

Response: We agree with the reviewer and added a couple of sentences in the limitations section to acknowledge this issue and how we used other data types as validation (Lines **425** - **435**).

“Another limitation of the model is the quality of the data used to calibrate the model. We focused on daily number of deaths because death reports are more reliable than case data. Nonetheless, the number of deaths in the city can also be underreported as it has been estimated in other countries[1]. To increase the reliability of our model calibration, we validated the model to other data types not included in the calibration, such as the infection attack rate”

3. *The section on Results could be more focused. I understand there is a variety of factors affecting the outcome of school reopening, but perhaps the authors should try to find a common thread though the various scenarios. This could possibly be centered around Figures 4 and 5, for instance.*

   Response: We addressed this comment by re-structuring the results and focusing on the outcomes suggested by the reviewer. We condensed the sections related to additional outcomes.

4. *The Discussion section is even less structured. Especially from Line 273 to Line 348, the presentation is a stream of short remarks on modeling choices and possible shortcomings that ends up becoming a little confusing. Some of the statements do actually refer to limitations more than discussion. Perhaps this part could be streamlined by moving some remarks to limitations, and adding perhaps a table summarizing the selected parameters.*

   Response: We thank the reviewer for the suggestion. We have now revised the discussion section (lines **274** - **376**) and moved the necessary parts to the limitation section.

5. *Line 25: perhaps it should be "priority in fighting poverty"*

   Response: We agree with the reviewer and made the suggested change (line **12**).

6. *Line 273: transmision - > transmission*

   Response: We have rewritten this paragraph.

7. *Line 289: christmas -> Christmas*

   Response: We made this correction in line **334**.

8. *Line 294: the sentence "A third wave in Bogotá, related to school opening is related to both, an increase in mixing patterns within schools and an accompanying increase in community transmission outside school" is not clear.*

   Response:

9. *Line 304: " level of poverty of schools attending the school" ??*

   Response: We made this correction (line **353**).

10. *Line 306: being -> having been*

    Response: We made this change in line **360**.

11. *Line 307: the sentence "Another factor associated with the small difference across MPI values is that schools attract few students from various neighborhoods around the city, increasing the probability that schools located in neighborhoods with low transmission could have imported infections from neighborhoods with high transmission" is not clear.*

    Response:

12. *Line 312: However -> However,*

    Response: We fixed this (line **386**)

13. *Line 316: "although" does not make sense here.*

    Response: We have made the suggested correction (line **389**).

14. *Lines 421, 422: increases vs. increased (there is no time coordination)*

    Response: We have made this change (line **514**.

15. *Line 438: (aOR: 0.73) ??*

    Response:

16. *Line 464 and others: prek -> pre-K*

    Response: We have made this change in the manuscript.

References

[1] Ariel Karlinsky and Dmitry Kobak. "Tracking Excess Mortality across Countries during the COVID-19 Pandemic with the World Mortality Dataset". In: *eLife* 10 (June 2021). Ed. by Miles P Davenport et al., e69336. ISSN: 2050-084X. DOI: 10.7554/eLife.69336.
