# Supplementary material for: Designing school reopening in the COVID-19 pre-vaccination period in Bogotá, Colombia: A modeling study
Source: PLOS Glob Public Health. 2022 Jun 15;2(6):e0000467. doi: 10.1371/journal.pgph.0000467 (PMC10021412; doi:10.1371/journal.pgph.0000467)
Supplement: Attachment — Submitted filename: manuscript_bogota_school_reopening_response_to_reviewers_R2.docx [file pgph.0000467.s003.docx]

# Response to Reviewers

We thank the reviewers for their thoughtful and constructive comments. We have addressed each of the comments and have elaborated a response for them. Please see below our point by point response for each of the reviewers’ concerns with line numbers referring to the PDF version of the report with changes marked in blue(additions) and red(deletions).

# Journal requirements

1. *Please provide us with a direct link to the base layer of the map used in Figure S2, Figure S5, and Figure S8, and ensure this location is also included in the figure legend.*

- **Response:**
- The base layer of the map used in these figures can be found in: <https://datosabiertos.bogota.gov.co/dataset/sector-catastral>.

# Reviewer 2

1. *Sure, the models are agent based: the word "compartmental" was a typo in the report. Still, I think that some of my comment there was appropriate, especially the part about the instability in the effect of school policies which seems to appear in the results presented in this paper.*

- **Response:**
- We have included in the section of limitations the following (Lines 265 - 274): “The evaluation of the impact on COVID-19 dynamics caused by school reopening depends on the epidemiological context. Hence, the predicted effectiveness of interventions to reduce transmission will often depend on whether the intervention reduces the reproduction number below 1, which can be sensitive to the model’s parameters . This effect means that, for example, the level at which school reopening capacity is optimized can be difficult to precisely quantify. Our qualitative results should, however, be robust to this effect, and we further mitigate it by exploring a range of scenarios. A caveat to this is that in our calibration, the reproduction number with schools fully opened was substantially greater than 1; if instead, the calibration led to a reproduction number below 1 with schools fully opened, then the impact of school closures would clearly be substantially reduced.”

2. *I might have missed it, but I did not find a summary of what actually happened in the period February-August 31, 2021: were schools closed? What was the observed mortality?*

- **Response:**
- We have added a summary of what happened in schools in lines 258 - 264. “Our study was set in January 2021 to understand the potential impact of school reopening. Although several other factors have affected the course of COVID-19 in the city, our study focuses on the effect of school transmission in the local context. In total, 16,000 deaths were reported in the period of February 2021 - August 2021 with schools partially opened and operating at lower capacity than other activities in the city. Although the magnitude of the third wave was higher than our scenarios, the magnitude of this wave has been attributed to the circulation of the ’mu’ variant , which was not included in this study.”

3. *Years are not shown in the figures. I guess Figure 1 is for 2020 and Figure 2 and 4 are for 2020 and part of 2021.*

- **Response:**
- We updated the manuscript figures to include years.
